# Macrophage Mistargeting During Direct Cardiac Reprogramming: Cardiac-Associated Responses, Polarization, and Cell Death

**DOI:** 10.3390/biomedicines14092089

**Published:** 2026-09-16

**Authors:** Tingzhen Chen, Shanshan Ding, Zhongwen Zhang, Lin Zhao, Kejun Cao, Guannan Zhang, Changya Weng, Guangyuan Gong, Liang Xu, Min Yang, Tianquan Li, Chengyu Sheng, Yanxiu Li

**Affiliations:** 1 Jiangsu Key Laboratory of Neurodegeneration, Nanjing Medical University, Nanjing 211166, China; 2 Department of Critical Care Medicine, The First Affiliated Hospital of Nanjing Medical University, Nanjing 210009, China; 3 School of Basic Medical Sciences, Nanjing Medical University, Nanjing 211166, China; 4 Department of General Surgery, The Affiliated Jiangning Hospital of Nanjing Medical University, Nanjing 211000, China; 5 Department of Critical Care Medicine, Chongqing Hospital of Jiangsu Province Hospital, Chongqing 401420, China; 6 Department of Emergency Medicine, Chongqing Hospital of Jiangsu Province Hospital, Chongqing 401420, China; 7 Center of Critical Care Medicine, The First Affiliated Hospital of Nanjing Medical University, Nanjing 210009, China; 8 Department of Emergency Medicine, The First Affiliated Hospital of Nanjing Medical University, Nanjing 210009, China

**Keywords:** direct cardiac reprogramming, MGT transcription factors, BMDMs, macrophage polarization, apoptosis, necroptosis

## Abstract

**Background/Objectives**: In situ reprogramming of cardiac fibroblasts into induced cardiomyocytes (iCMs) holds great promise for myocardial infarction (MI) therapy by replenishing lost cardiomyocytes. A transcription factor cocktail consisting of Mef2c, Gata4, and Tbx5 (MGT) represents a widely used starting regimen for cardiac reprogramming, with numerous optimization efforts focused primarily on improving reprogramming efficiency. However, the effects of MGT exposure on non-target cardiac cell populations remain largely uncharacterized, and it remains unclear whether such unintended effects could pose safety concerns. **Methods**: In this study, we introduced the widely used MGT cocktail into primary mouse bone marrow-derived macrophages (BMDMs) and the immortalized mouse macrophage cell line RAW 264.7. We first assessed cardiomyocyte-associated gene and protein expression. We then examined the polarization state of transduced RAW 264.7 cells. In BMDMs, we further assessed apoptosis- and necroptosis-associated markers to determine whether MGT delivery was accompanied by cell death. **Results**: (a) MGT introduction induced a transient increase in cardiomyocyte-associated Tnnt2/Myh6 transcripts and modest cTnT protein induction in macrophages, with cTnT immunofluorescence remaining elevated after two weeks in BMDMs and a small subset of cells displaying fibroblast-like morphology reminiscent of iCMs. (b) Transduced RAW 264.7 cells displayed elevated Cd206 transcript levels, accompanied by an increased proportion of spindle-like cells and enlarged nuclear area, collectively supporting an M2-like polarization shift. (c) Transduced primary BMDMs, but not RAW 264.7 cells, displayed elevated apoptosis- and necroptosis-associated responses, including increased YP1 fluorescence, PI staining, and p-MLKL immunofluorescence. **Conclusions**: The classical MGT reprogramming regimen polarized RAW 264.7 cells toward an M2-like state, raising the possibility that, in the TGF-β-rich post-MI environment, this shift could increase the pool of macrophages susceptible to macrophage-to-myofibroblast transition and thereby potentially promote fibrosis. MGT-expressing LV elicited apoptosis-associated and necroptosis-associated changes in transduced BMDMs. These polarization and cell-survival effects highlight potential off-target and safety concerns for in situ direct cardiac reprogramming. Further investigations are warranted to determine whether and how such macrophage perturbations affect tissue remodeling and therapeutic outcomes in the post-MI setting.

## 1. Introduction

Cardiovascular diseases (CVDs) continue to pose a formidable challenge to global public health, accounting for approximately one-third of all deaths worldwide [1,2]. Heart failure is a major consequence of CVDs and is primarily associated with the loss of cardiomyocytes or dysfunction of existing cardiomyocytes [1]. Despite significant advances in surgical interventions and cardiac transplantation, these approaches remain cost-prohibitive and are severely limited by a shortage of donor hearts [3]. The human heart has a limited capacity to regenerate cardiomyocytes. In 2010, Ieda et al. reported the successful generation of mouse cardiomyocyte-like cells from postnatal cardiac or dermal fibroblasts using a combination of three transcription factors, Mef2c, Gata4, and Tbx5 (collectively referred to as MGT) [4]. Subsequently, direct lineage reprogramming of cardiac fibroblasts (CFs) into functional cardiomyocytes has attracted major attention as a strategy for heart regeneration. Over the past decade, researchers have substantially improved conversion efficiency by either introducing additional transcription factors, adding small molecules that target specific cellular pathways, or employing new delivery vehicles such as Sendai viral vectors [5,6,7,8,9]. While these efforts have substantially advanced cardiac reprogramming, they have focused predominantly on CFs and have largely overlooked the potential off-target effects of reprogramming factors and associated interventions on other cardiac-resident cell types. To our knowledge, the present study represents the first systematic investigation of the consequences of MGT exposure in macrophages as a non-target cell population during cardiac reprogramming.

The MGT combination remains the cornerstone of most direct cardiac reprogramming strategies. These transcription factors are well-established regulators of cardiac development and cardiomyocyte identity, and their downstream programs include genes involved in contractile function and calcium handling, such as the sarcomeric genes *Myh6* and *Tnnt2* and the ion channel genes *Ryr2* and *Scn5a* [10]. The rationale for this combination is rooted in developmental biology, as Gata4, Mef2c, and Tbx5 each play essential roles in cardiac specification, myocardial development, and cardiomyocyte maturation, respectively [11,12,13,14].

The ultimate goal of direct conversion is in situ reprogramming, in which resident cardiac fibroblasts are converted into cardiomyocytes within the injured heart. Because cardiac fibroblasts naturally coexist with cardiomyocytes in the same niche, this strategy may offer advantages for tissue integration compared with conventional cell transplantation. However, in situ delivery of reprogramming reagents also creates the possibility of unintended exposure of other cardiac cell populations. Besides cardiac fibroblasts, other cell types may be exposed to reprogramming factors, particularly when transcription factors or small molecules lacking strict cell-type specificity are used. Among these potential off-target populations, macrophages warrant particular attention because both resident and infiltrating macrophages are abundant and functionally important in the post-infarction myocardium [15]. Viral vectors such as lentiviruses (LVs) may also be taken up by macrophages, and even when cell-type-specific promoters are employed, unintended ectopic expression in off-target cells cannot be completely excluded. This concern may be further increased when small-molecule compounds are used for in vivo reprogramming, as these compounds generally lack cell-type-specific targeting. Given the important roles of macrophages in inflammation, angiogenesis, tissue repair, and cardiomyocyte proliferation [16], unintended perturbation of their state or survival could have important consequences for cardiac repair. To address this potential mistargeting concern, we investigated the responses of primary bone marrow-derived macrophages (BMDMs) and the RAW264.7 macrophage cell line to the classical MGT reprogramming cocktail. We examined cardiac-associated gene expression, macrophage polarization, and cell-death-associated responses, with a focus on apoptosis and necroptosis.

## 2. Materials and Methods

### 2.1. Cell Culture

Bone marrow cells were isolated from the femurs and tibias of 8-week-old C57BL/6J mice by flushing the bone marrow with Dulbecco’s Modified Eagle Medium (DMEM). After 4 h of incubation, adherent cells were removed and non-adherent cells were collected and differentiated into BMDMs over 7 days in DMEM containing 10% fetal bovine serum (FBS, Gibco, NY, USA, 10099-141C), 1% penicillin–streptomycin (P-S, Gibco), and 20 ng/mL macrophage colony-stimulating factor (M-CSF, Peprotech, NJ, USA, 315-02), with fresh medium added on day 3. Alternatively, bone marrow cells were differentiated into BMDMs by culture in complete RPMI 1640 medium containing L929-conditioned medium for one week [17]. The RAW264.7 macrophage cell line was maintained in complete RPMI 1640 supplemented with 10% FBS and 1% P-S. Both BMDMs and RAW264.7 cells were prepared for subsequent experiments.

To obtain CFs, cardiomyocytes and non-myocytes (including CFs and endothelial cells) were isolated from the hearts of 0–3-day-old neonatal C57BL/6J mice. Then, CFs were sorted out by using the Neonatal Heart Dissociation Kit (Miltenyi Biotec, Bergisch Gladbach, Germany, 130-098-373), enriched by differential adhesion and cultured in growth medium consisting of IMDM with sodium pyruvate and L-glutamine (Procell, Wuhan, China, PM150510) supplemented with 20% FBS and 1% P-S; the medium was changed every three days, and cells were maintained at 37 °C with 5% CO_2_ as previously described [18]. Once fibroblasts reached 70–80% confluence, they were prepared for use.

### 2.2. Plasmids and Transfection

The plasmid encoding Mef2c, Gata4 and Tbx5 was sourced from the pGEMT-TPE2A-Mef2c-Tdtomato-Gata4-Tbx5 (Addgene #111818). The coding regions of Gata4, Mef2c, and Tbx5 were amplified by PCR and subcloned into a lentiviral vector backbone—pLV hUbC-dCas9 KRAB-T2A-GFP (Addgene #67620). These coding regions were then further subcloned into the lentiviral vector backbone—pLVX-IRES-Puro (from the Lenti-X™ Bicistronic Expression System (Puro), Cat. No. 632183)—to generate a polycistronic construct that encodes all three factors in a single mRNA (termed “MGT”).

For plasmid transfection, cells were transfected with the MGT plasmid using Lipofectamine™ 3000 (Invitrogen, Carlsbad, CA, USA, L300075), and the transfection was repeated every 84 h. At 84 h after the first transfection or after transduction, the medium was replaced with iCM medium composed of DMEM/M199 (4:1) supplemented with 10% FBS, 1% non-essential amino acids (NEAA), and 1% P-S. Thereafter, the medium was changed every 72 h, and cells were maintained for one or two weeks [19,20,21].

### 2.3. Virus Transduction

LV encoding the polycistronic construct MGT and empty vector LV were packaged and produced by OBiO Technology, Shanghai, China. At 48 h post-transduction, the virus-containing medium was replaced with iCM medium and changed every 2–3 days for one week or two weeks.

### 2.4. Quantitative Real-Time PCR (qPCR)

Total RNA was isolated using RNAiso Plus (Takara Bio, Kusatsu, Shiga, Japan). Reverse transcription was performed with a cDNA reverse transcription kit (EZB-RT2GQ, EZBioscience, Shanghai, China), and quantitative real-time PCR analysis was subsequently carried out using SYBR Green qPCR Master Mix (EZB-A0012-R1). The mRNA expression levels were normalized to GAPDH. The qPCR primers used are listed in Table S1.

### 2.5. Western Blotting (WB)

Cells were lysed in RIPA buffer (Beyotime, Shanghai, China, P0013B) supplemented with a protease inhibitor cocktail (Beyotime, ST506) and PMSF (Roche, Basel, Switzerland, 0490683701). Protein concentration was determined using the Enhanced BCA Protein Assay Kit (Beyotime, P0009). Equal amounts of protein were separated by SDS-PAGE and transferred onto PVDF membranes (Millipore, Billerica, MA, USA, 0.2 µm, ISEQ00010). The membranes were blocked with 5% nonfat milk and incubated overnight at 4 °C with primary antibody against Tbx5 (ZenBio, Chengdu, China, 861904) or cTnT (Proteintech, Rosemont, IL, USA, 26592-1-AP). After washing, the membranes were probed with HRP-conjugated secondary antibody for 1 h at room temperature. Protein bands were detected with High-sig ECL Substrate (Tanon, Shanghai, China, 180-5001). Relative bands were quantified using NIH ImageJ 1.54 Software.

### 2.6. Immunocytochemistry (ICC)

For immunofluorescence staining, cells cultured on glass coverslips were treated with 4% paraformaldehyde (PFA) and washed with PBS. The samples were permeabilized with PBS containing 0.01% Triton X-100 for 10 min and then blocked with 5% BSA in PBS for 1 h to reduce nonspecific antibody binding. After blocking, the cells were incubated overnight at 4 °C with primary antibodies for double staining: Mef2c (Abcam, Cambridge, UK, ab211493) and cTnT (Santa Cruz Biotechnology, Dallas, TX, USA, sc-20025), or Gata4 (ZenBio, 381799) and cTnT. Following three washes with PBS, the cells were incubated with appropriate fluorescently conjugated secondary antibodies for 1–2 h at room temperature. All samples were counterstained with 4′,6-diamidino-2-phenylindole (DAPI) for 7 min and mounted with an anti-fade mounting medium. Fluorescence images were captured using an Olympus microscope (Olympus, Tokyo, Japan) and analyzed with ImageJ 1.54.

### 2.7. YO-PRO-1 (YP1) and PI Staining

MGT-treated cells were stained separately with YO-PRO-1 (YP1) and propidium iodide (PI) (1:1000 dilution) and incubated at 37 °C in the dark for 20 min. Images were acquired using a live-cell workstation (Zeiss Celldiscoverer 7, Zeiss, Oberkochen, Germany), and fluorescence intensities in the plates were quantified with a microplate reader. YP1-positive cells emitted green fluorescence (excitation/emission: 491/509 nm), while PI-positive cells emitted red fluorescence (excitation/emission: 535/617 nm). The level of apoptosis was determined based on the ratio of (YP1-positive − PI-positive) cells to Hoechst-positive cells.

### 2.8. Necroptosis Evaluation with p-MLKL

The phosphorylation of MLKL promotes its oligomerization and membrane translocation and is closely associated with necroptotic signaling. Immunofluorescence staining against p-MLKL was therefore performed to assess necroptosis-associated responses. For quantitative analysis, all captured immunofluorescence images were processed with an identical threshold setting. The proportion of cells with p-MLKL-positive puncta relative to the total cell count was calculated in each microscopic field. Three independent fields were imaged and quantified per biological sample to generate an averaged ratio, with three biological replicates included for each experimental group.

### 2.9. Statistical Analysis

Data are presented as mean ± standard error of the mean (SEM). Continuous variables were compared with the Student’s t-test. All statistical analyses were performed with GraphPad Prism 6.0.6 for Windows, GraphPad Software, San Diego, CA, USA, www.graphpad.com. All “*n*” values reported in the statistical analyses represent independent biological replicates and do not refer to technical replicates. *p* < 0.05 was considered statistically significant. * *p* < 0.05, ** *p* < 0.01, *** *p* < 0.001 and **** *p* < 0.0001.

## 3. Results

### 3.1. MGT Induces Cardiomyocyte-Associated Gene Expression in Mouse CFs

**Figure 1:**
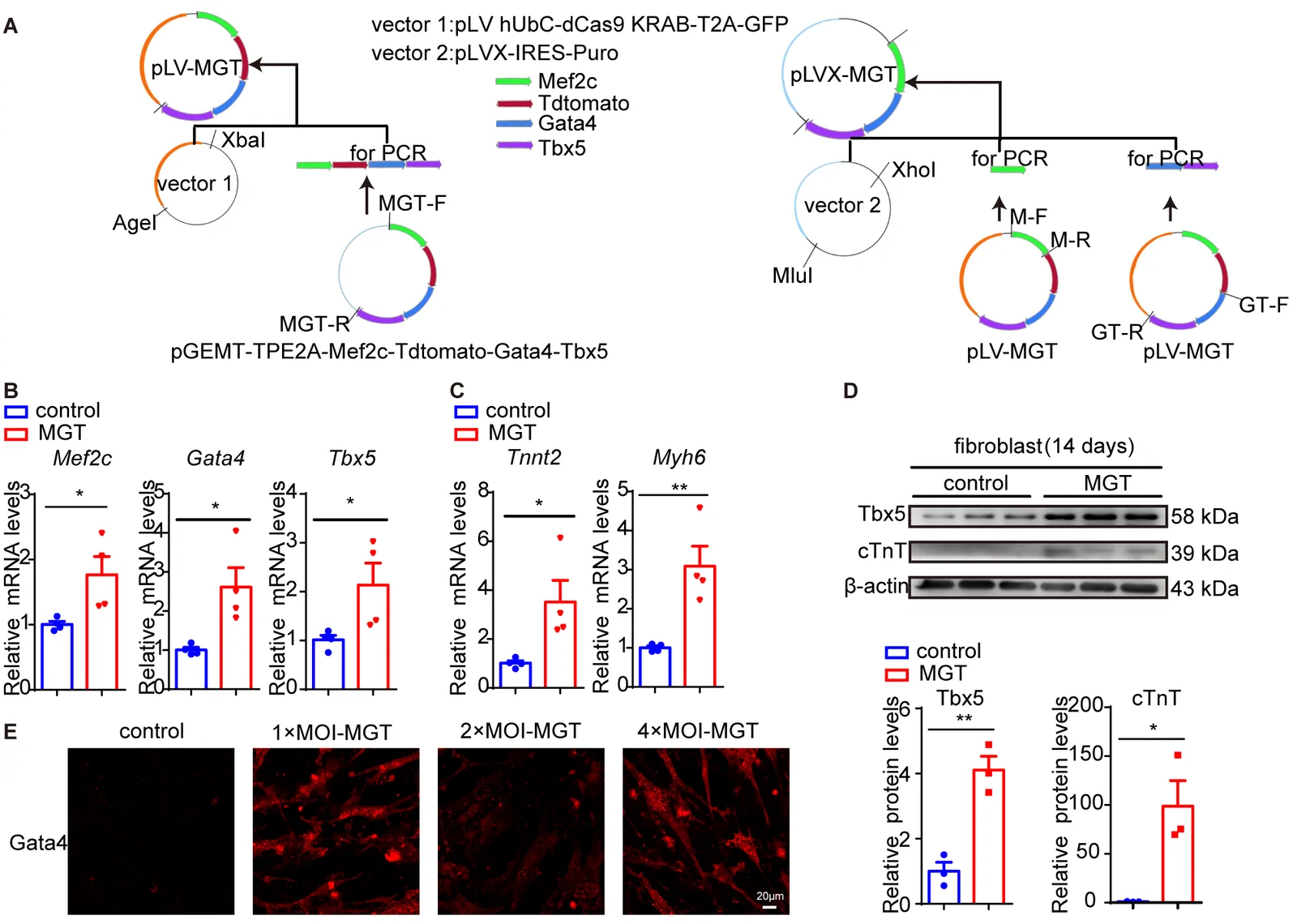
MGT induces cardiomyocyte-associated gene and protein expression in mouse CFs. (**A**) Experimental design for evaluating the MGT polycistronic lentiviral construct in CFs. CFs were transfected with the lentiviral plasmid for one week (**B**,**C**) or transduced with the corresponding lentiviral particles for two weeks (**D**,**E**). (**B**,**C**) Bar graphs (Mean + SEM) with overlaid dot plots (individual data points) show the corresponding mRNA levels of *Mef2c*, *Gata4*, *Tbx5*, *Tnnt2*, and *Myh6* evaluated by qPCR (n = 4). (**D**) Protein levels of Tbx5 and cTnT in CFs transfected with or without MGT lentiviral plasmid (n = 3). Bar graphs (Mean + SEM) with overlaid dot plots (individual data points) show the corresponding quantification of Tbx5 and cTnT levels in (**D**). (**E**) Immunofluorescence staining of Gata4 (protein, shown in red) in CFs transduced with LV at 1× (MOI = 50), 2× (MOI = 100), and 4× MOI (MOI = 200). Scale bar = 20 μm. * *p* < 0.05, ** *p* < 0.01. Unpaired Student’s *t*-test.

Since high conversion efficiency depends on the proper stoichiometry of Gata4, Mef2c, and Tbx5, with greater Mef2c expression required than that of Gata4 or Tbx5 (with the MGT arrangement shown to be more effective than MTG, GTM, GMT, TMG, or TGM in previous studies) [22], we constructed a lentiviral vector expressing MGT as a polycistronic construct to ensure an optimal expression ratio. The MGT polycistronic construct was generated as illustrated in Figure 1A. Successful transduction and expression of the three factors were validated by qPCR analysis of their respective mRNA levels 7 days after transfection of cultured primary CFs with the lentiviral plasmid (Figure 1B). Concurrently, the transcript levels of *Tnnt2* and *Myh6* were significantly increased (Figure 1C), indicating induction of cardiomyocyte-associated genes in the transduced CFs. To further substantiate these findings, we packaged the lentiviral plasmid to produce lentiviral particles and transduced CFs, extended the treatment period to two weeks, and examined the protein expression of Tbx5 and cTnT. Western blotting revealed markedly elevated protein levels of both Tbx5 and cTnT in the transduced group, further supporting MGT-induced cardiomyocyte-associated response by MGT (Figure 1D). Furthermore, we optimized the multiplicity of transduction (MOI) for subsequent experiments by performing immunofluorescence analysis on fibroblasts transduced with the corresponding packaged LV at 1× (MOI = 50), 2× (MOI = 100), and 4× MOI (MOI = 200). MOI = 50 was selected as the optimal dose for subsequent experiments based on Gata4 expression (Figure 1E).

### 3.2. One-Week MGT Treatment Induces a Cardiomyocyte-Associated Molecular Response in RAW264.7 Cells

**Figure 2:**
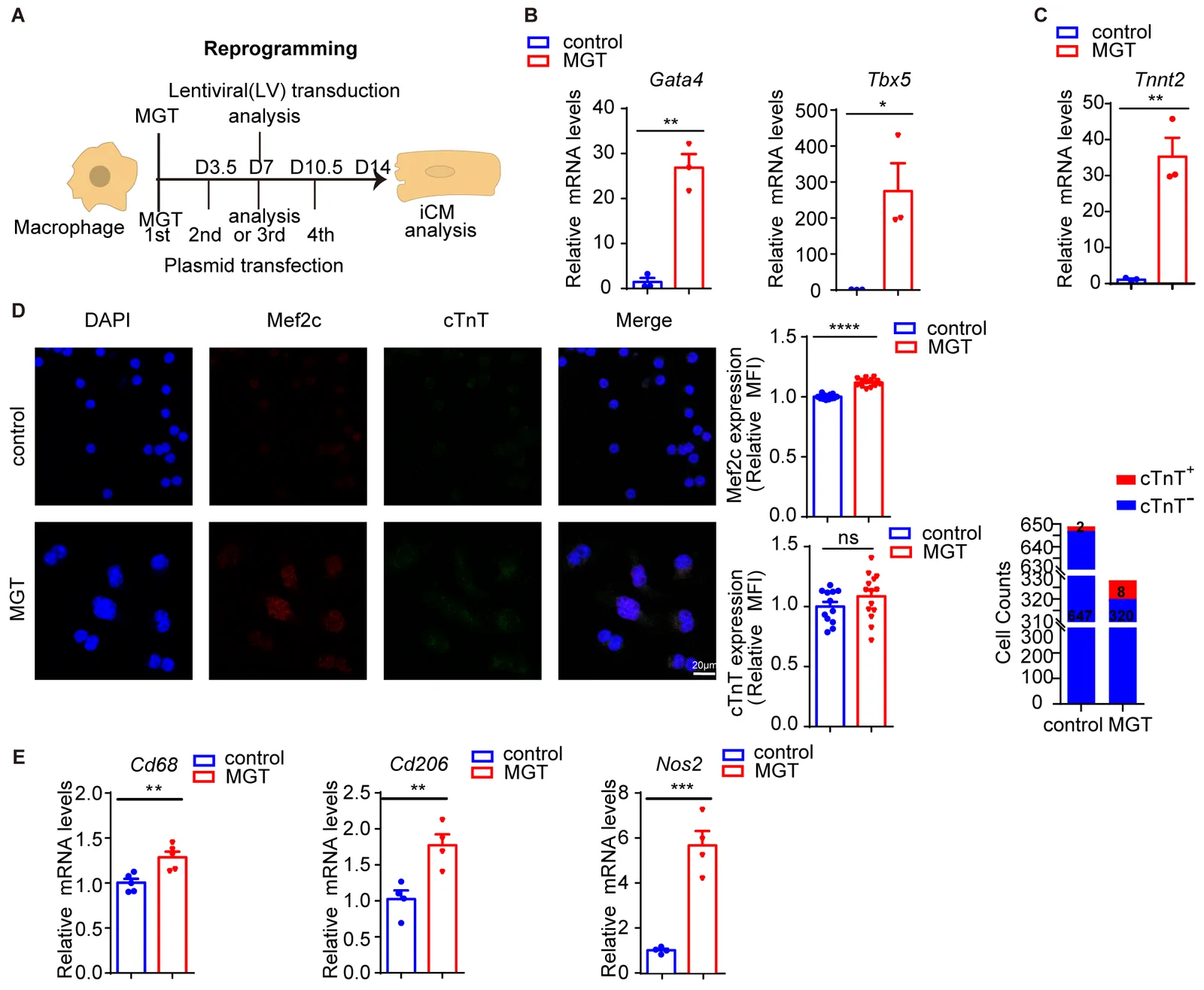
One-week MGT treatment induces a partial cardiac-associated response and alters macrophage polarization gene expression in RAW264.7 cells. (**A**) Schematic illustration of the experimental design for introducing MGT into macrophages using the indicated lentiviral plasmid or the corresponding packaged LV. (**B**,**C**) RAW264.7 cells were transduced with the LV delivering MGT for one week. Bar graphs (Mean + SEM) with overlaid dot plots (individual data points) show the mRNA levels of *Mef2c*, *Gata4, Tbx5, Tnnt2,* and *Myh6* evaluated by qPCR (n = 3). (**D**) Immunofluorescence co-staining for Mef2c and cTnT in RAW264.7 cells with or without the lentiviral plasmid transfection overexpressing MGT for one week (n = 12–14). Blue: DAPI staining of nucleus; Red: Mef2C staining; Green: cTnT staining. Right: bar graphs (Mean + SEM) with overlaid dot plots show the mean fluorescence intensity (MFI) of Mef2c and cTnT, and the cell counts of cTnT^+^ and cTnT^−^ cells between two groups. Scale bar = 20 μm. (**E**) Bar graphs (Mean + SEM) with overlaid dot plots show the mRNA levels of *Cd68*, *Nos2* and *Cd206* evaluated by qPCR in RAW264.7 cells with or without the lentiviral plasmid transfection overexpressing MGT for one week (n = 4). Error bars represent Mean + SEM. * *p* < 0.05, ** *p* < 0.01, *** *p* < 0.001, **** *p* < 0.0001. ns, not significant. Unpaired Student’s *t*-test.

Next, we cultured RAW264.7 cells, a widely used macrophage cell line isolated from a mouse tumor, and investigated the effects of MGT introduction (Figure 2A). With the same MGT-expressing LV, qPCR analysis revealed significantly elevated transcript levels of *Gata4* and *Tbx5* one week after transduction (Figure 2B), indicating successful gene delivery and expression. Interestingly, as in primary CFs, we found that MGT introduction markedly elevated the mRNA level of *Tnnt2* (Figure 2C). Given that Mef2c is a known direct activator of *Tnnt2* [23,24], this finding suggests that the *Tnnt2* locus is responsive to MGT expression in RAW264.7 cells and raises the possibility of a partial cardiomyocyte-associated transcriptional response. To further investigate this possibility, we transfected RAW264.7 cells with the MGT-encoding lentiviral plasmid for one week and examined cTnT protein levels. Immunofluorescence analysis detected cTnT^+^ cells in both groups, with a higher proportion observed in the MGT group (8 out of 328, 2.44%) than in the control group (2 out of 649, 0.31%) (Figure 2D). cTnT immunofluorescence intensity showed a trend toward elevation in Mef2c-positive cells, although the difference did not reach statistical significance (Figure 2D). No overt cardiomyocyte-like morphological changes or contractile phenotype were observed in MGT-transfected cells, although a reduction in cell number was evident in the MGT group. We next examined macrophage polarization, focusing on M1 and M2 phenotypic states. One week after MGT plasmid transfection, qPCR analysis revealed upregulated mRNA expression of the pan-macrophage marker *Cd68*, as well as both M1 (*Nos2*) and M2 (*Cd206*) marker genes in RAW264.7 cells (Figure 2E). These findings suggest an early broad alteration of macrophage activation, with both M1- and M2-associated genes upregulated following MGT introduction.

### 3.3. Two-Week MGT Treatment Induces Fibroblast-Like Morphological Changes and an M2-Like Polarization-Associated Response in RAW264.7 Cells

**Figure 3:**
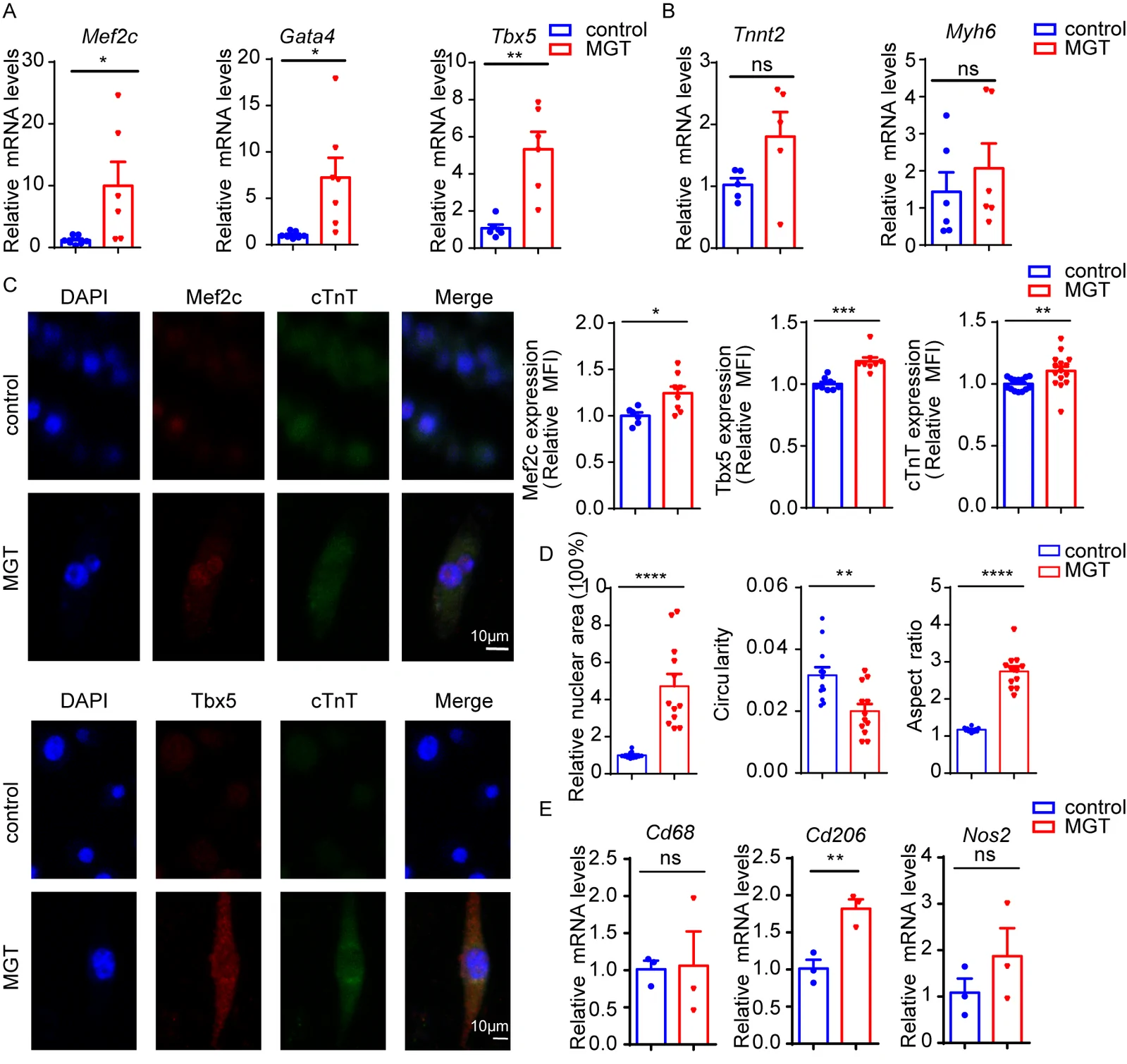
Two-week MGT plasmid transfection induces a partial cardiac-associated response and an M2-like polarization-associated phenotype in RAW264.7 cells. (**A**,**B**) Bar graphs (Mean + SEM) with overlaid dot plots (individual data points) show the corresponding mRNA levels of *Mef2c* (n = 6–7), *Gata4* (n = 7), *Tbx5* (n = 6), *Tnnt2* (n = 5, *p = 0.0955*), and *Myh6* (n = 6) evaluated by qPCR. (**C**) Immunofluorescence co-staining for Mef2c and cTnT (n = 6–8) or Tbx5 and cTnT (n = 8) in RAW264.7 cells transfected with or without the MGT plasmid for two weeks. Blue: DAPI staining of nucleus; Red: staining of Mef2C (the upper panel) or Tbx5 (the lower panel); Green: cTnT staining. Scale bar = 10 μm. Right panels: bar graphs (Mean + SEM) with overlaid dot plots (individual data points) show the relative MFI of Mef2C, Tbx5, and cTnT. (**D**) Bar graphs (Mean + SEM) with overlaid dot plots (individual data points) show the nuclear area, circularity, and aspect ratio of cells quantified from immunofluorescence images shown in (**C**) (n = 12). (**E**) Bar graphs (Mean + SEM) with overlaid dot plots (individual data points) show the corresponding mRNA expression of *Cd68*, *Nos2* and *Cd206* evaluated by qPCR (n = 3). * *p* < 0.05, ** *p* < 0.01, *** *p* < 0.001, **** *p* < 0.0001. ns, not significant. Unpaired Student’s *t*-test.

To determine whether the response to MGT treatment progressed with extended exposure, we prolonged the MGT plasmid transfection to two weeks. Analysis with qPCR confirmed that the mRNA levels of *Mef2c*, *Gata4*, and *Tbx5* remained significantly elevated (Figure 3A). However, the transcript levels of the cardiomyocyte-associated genes *Tnnt2* and *Myh6* showed only an increasing trend without reaching statistical significance (Figure 3B). These findings indicate that MGT treatment induces a persistent cardiomyocyte-associated molecular response in RAW264.7 cells, although evidence for cardiomyocyte conversion remains limited. At the protein level, this extended MGT treatment led to a mild but significant increase in cTnT immunofluorescence intensity (Figure 3C). Interestingly, cells receiving MGT tended to exhibit significantly enlarged nuclei and a spindle-shaped morphology, closely resembling the characteristic appearance of M2 macrophages (Figure 3D). Consistent with this observation, the elevation of *Cd206* persisted after two weeks of MGT transfection, whereas the M1-associated *iNos* expression was no longer increased (Figure 3E), suggesting a shift toward an M2-like state over time.

### 3.4. One-Week MGT Treatment Induces a Cardiomyocyte-Associated Molecular Response in Primary BMDMs

**Figure 4:**
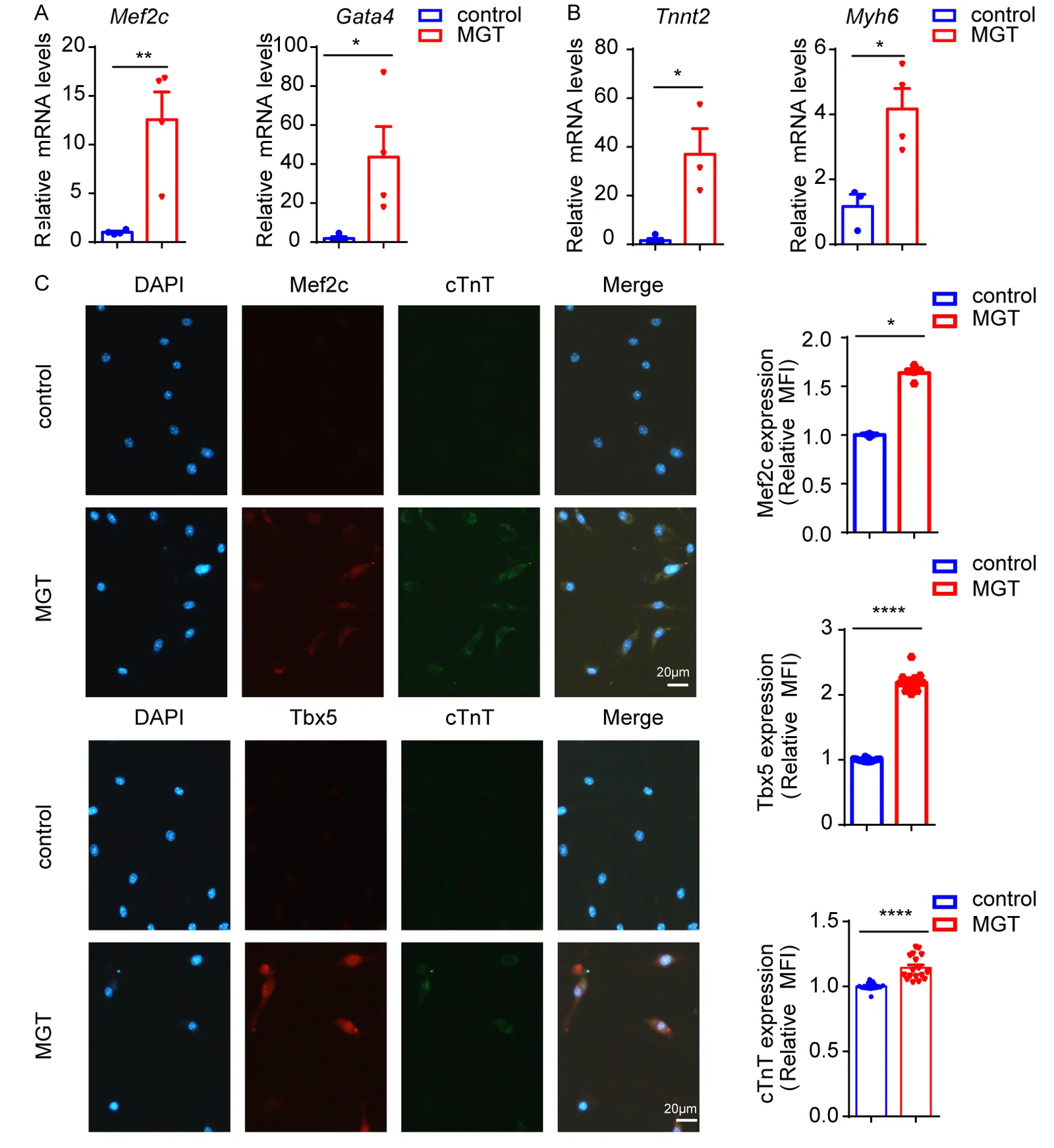
One-week MGT treatment induces a partial cardiac-associated response in primary BMDMs. (**A**,**B**) Primary BMDMs were transduced with MGT-expressing LV for one week. Bar graphs (Mean + SEM) with overlaid dot plots (individual data points) show the corresponding mRNA levels of *Mef2c*, *Gata4*, *Tbx5*, and *Tnnt2* evaluated by qPCR (n = 3–4). (**C**) Primary BMDMs were transfected with the MGT-expressing plasmid for one week. Immunofluorescence co-staining for Mef2c and cTnT or Tbx5 and cTnT (n = 15–17). Blue: DAPI staining of nucleus; Red: staining of Mef2C (the upper panel) or Tbx5 (the lower panel); Green: cTnT staining. Right: bar graphs (Mean + SEM) with overlaid dot plots (individual data points) show the relative MFI of Mef2C, Tbx5, and cTnT (combined). Scale bar = 20 μm. * *p* < 0.05, ** *p* < 0.01, **** *p* < 0.0001. Unpaired Student’s *t*-test.

RAW264.7 is an immortalized mouse macrophage cell line that may behave differently from primary macrophages. We therefore cultured primary BMDMs and subjected them to lentiviral transduction or MGT plasmid transfection. One week after transduction with MGT-expressing LV, we detected significantly increased mRNA levels of *Mef2c* and *Gata4* (Figure 4A), confirming successful gene delivery and expression. Consistent with the findings in RAW264.7 cells, the transcript levels of *Tnnt2* and *Myh6* were also significantly elevated (Figure 4B). At the protein level, immunofluorescence analysis showed a significant increase in cTnT immunofluorescence intensity following MGT plasmid transfection in Mef2c^+^ and Tbx5^+^ cells (Figure 4C). Moreover, modest spindle-like morphological changes were observed in the MGT group.

### 3.5. A Small Subset of BMDMs Display Fibroblast-Like Morphology Reminiscent of iCMs After Two-Week Lentiviral MGT Transduction

**Figure 5:**
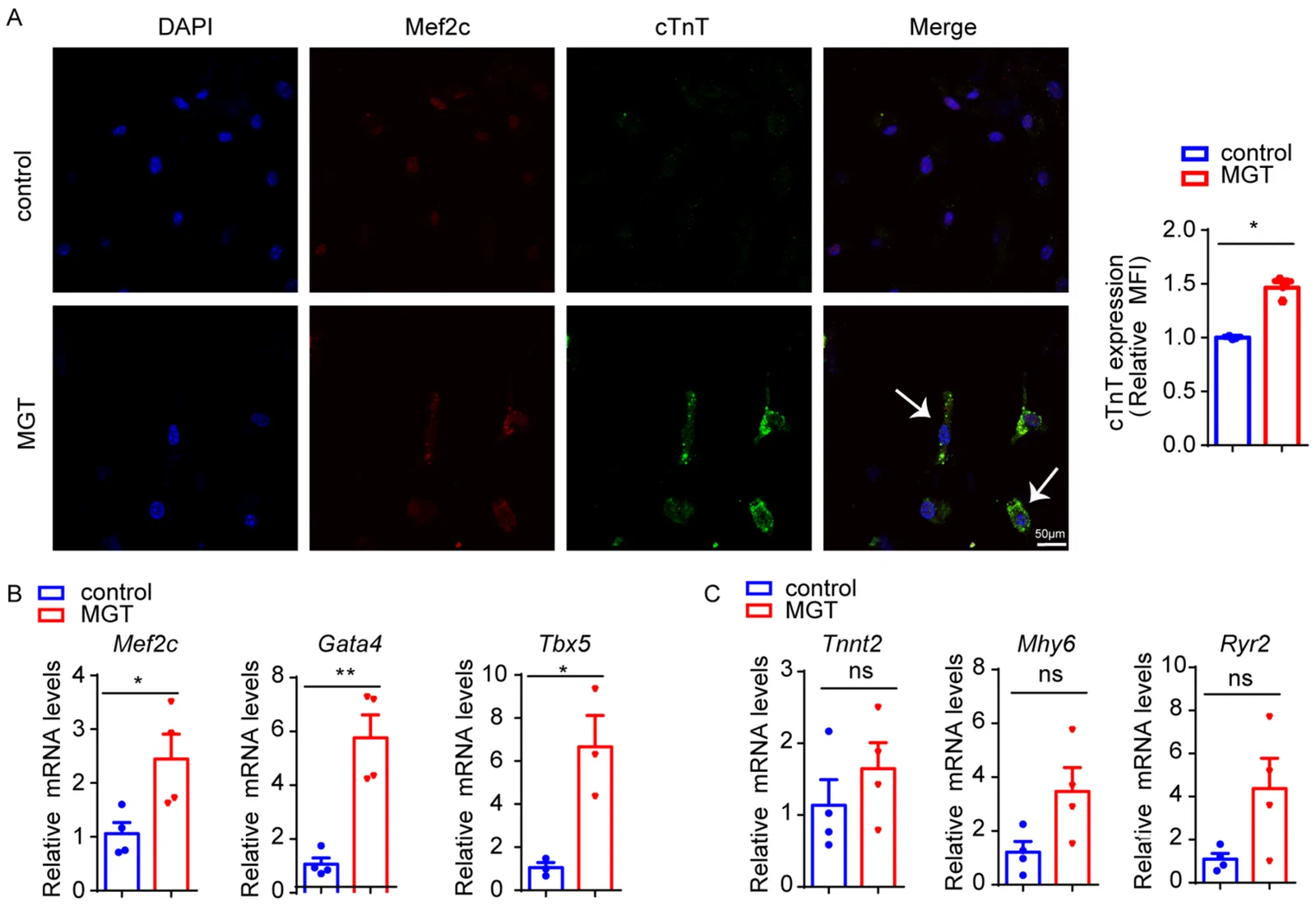
Two-week lentiviral MGT transduction induces fibroblast-like morphological changes accompanied by a partial cardiac-associated response in a small subset of BMDMs. (**A**) Immunofluorescence co-staining for Mef2c and cTnT; cTnT^+^ cells exhibit a fibroblast-like morphology. Blue: DAPI staining of nucleus; Red: Mef2C staining; Green: cTnT staining. White arrows point to two representative cells. Right: bar graphs (Mean + SEM) with overlaid dot plots (individual data points) show the relative MFI of cTnT. Scale bar = 50 μm. (**B**,**C**) Bar graphs (Mean + SEM) with overlaid dot plots show the mRNA levels of *Mef2c*, *Gata4*, *Tbx5*, *Tnnt2*, *Myh6*, and *Ryr2* (n = 3–4). * *p* < 0.05, ** *p* < 0.01. ns, not significant. Unpaired Student’s *t*-test.

Since the one-week MGT regimen produced a trend of increased cTnT expression, particularly in Mef2c^+^ and Tbx5^+^ cells, we extended the period to two weeks with lentiviral transduction. Consistent with this trend, we observed higher cTnT immunofluorescence intensity in Mef2c^+^ cells compared with control BMDMs receiving empty vector LV (Figure 5A); moreover, a small subset of cells exhibited fibroblast-like morphological changes reminiscent of induced cardiomyocytes (iCMs) reported in previous studies [25] (Figure 5A). However, this phenotype should not be interpreted as evidence of successful conversion of BMDMs into a cardiomyocyte-like state. As mentioned above, M2-like macrophages can exhibit fibroblast-like morphology, and it is possible that the morphology of a small subset of cells may resemble that of iCMs.

At the mRNA level, similar to the results in RAW264.7 cells, qPCR revealed sustained elevation of *Mef2c*, *Gata4*, and *Tbx5* transcripts (Figure 5B). However, the transcript levels of the cardiomyocyte-associated genes *Tnnt2*, *Myh6*, and *Ryr2* all showed consistent trends toward elevation, although none reached statistical significance (Figure 5B,C). Together, these findings suggest that the cardiomyocyte-associated transcriptional response induced by MGT persists at two weeks, although it is less pronounced than at one week.

### 3.6. Lentiviral MGT Transduction Induces Apoptosis- and Necroptosis-Associated Responses in Primary BMDMs

**Figure 6:**
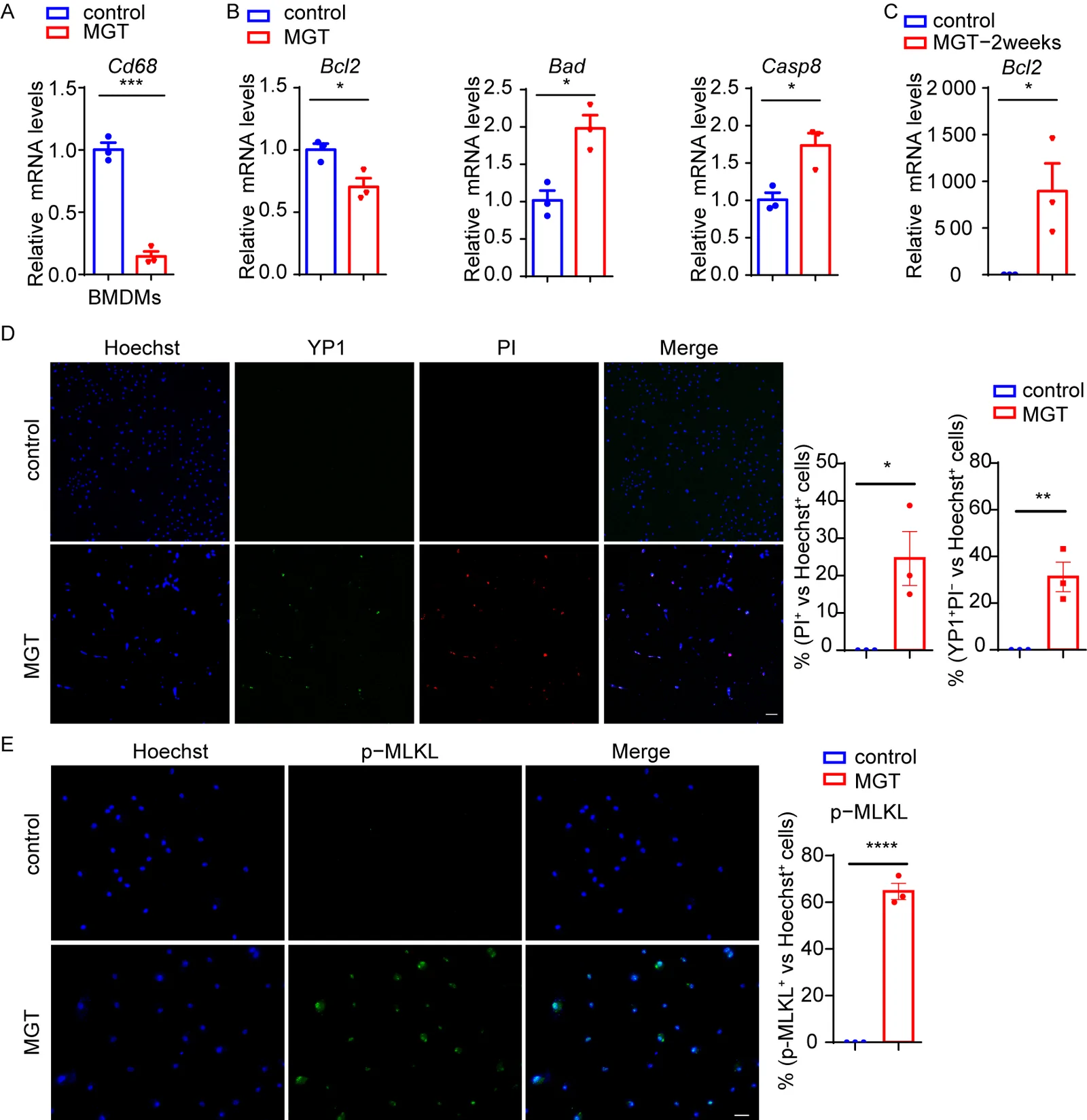
Lentiviral MGT transduction induces apoptosis- and necroptosis-associated responses in primary BMDMs. (**A**,**B**,**D**,**E**) BMDMs were transduced with MGT-expressing LV for one week. (**C**) BMDMs were transduced with MGT-expressing LV for two weeks. (**A**) *Cd68* mRNA levels assessed by qPCR (n = 3). (**B**) mRNA levels of *Bcl2*, *Bad*, and *Casp8* (n = 3). (**C**) *Bcl2* mRNA levels after two-week MGT LV treatment (n = 3). (**D**) YP1 and PI staining to assess cell-death-associated changes. Blue: Hoechst staining of nucleus; Green: YP1 staining; Red: PI staining. Right: bar graphs (Mean + SEM) with overlaid dot plots show the percentage of PI^+^ and YP1^+^PI^−^ cells. Scale bar = 20 μm. (**E**) Immunofluorescence staining for p-MLKL (n = 3), with p-MLKL-positive puncta quantified as a necroptosis-associated readout. Blue: Hoechst staining of nucleus; Green: p-MLKL staining. Right: bar graphs (Mean + SEM) with overlaid dot plots show the percentage of p-MLKL^+^ cells. Scale bar = 20 μm. * *p* < 0.05, ** *p* < 0.01, *** *p* < 0.001, **** *p* < 0.0001. ns, not significant. Unpaired Student’s *t*-test.

For the cell-death analyses, we used lentiviral transduction rather than plasmid transfection. After one week of MGT transduction, a significant decrease in *Cd68* mRNA levels was observed in primary BMDMs (Figure 6A). Bcl-2 family proteins regulate apoptotic cell survival, whereas caspase-8 is an important regulator of apoptotic and necroptotic cell-death pathways [26,27], with phosphorylated mixed lineage kinase domain-like protein (p-MLKL) serving as a key mediator of necroptosis [28,29]. We therefore evaluated apoptosis- and necroptosis-associated markers by qPCR and immunofluorescence following one week of MGT LV transduction. The results revealed significant downregulation of the anti-apoptotic gene *Bcl2* and marked upregulation of the pro-apoptotic genes *Bad* and *Casp8* (Figure 6B), suggesting activation of an apoptosis-associated response in BMDMs after one week of MGT treatment. In contrast, after two weeks of MGT treatment, BMDMs showed significant upregulation of *Bcl2* alongside sustained upregulation of *Bad* and *Casp8*, suggesting activation of an anti-apoptotic response despite persistent pro-apoptotic signaling (Figure 6C). YP1 is a green fluorescent dye that permeates the membranes of apoptotic cells, whereas propidium iodide (PI) stains necrotic cells with compromised membrane integrity, emitting red fluorescence. Thus, YP1^+^PI^−^ cells were considered consistent with apoptotic cells, whereas PI^+^ indicated loss of plasma membrane integrity. The elevation of the YP1^+^PI^−^ ratio further supported the presence of apoptosis-associated cell death after two weeks of MGT treatment (Figure 6D). In accordance with our results, a recent study reported that inducible MGT expression caused apoptosis in mouse embryonic fibroblasts [30], together raising the concern that aberrant MGT expression may compromise macrophage survival.

Similarly, the elevated PI^+^ ratio indicated increased loss of plasma membrane integrity, consistent with necrotic cell death (Figure 6D). Necroptosis is a form of regulated necrosis and shares stimuli and molecular machinery with apoptosis. We therefore examined p-MLKL, a key mediator of necroptosis, in transduced BMDMs. The proportion of p-MLKL^+^ cells was markedly increased at both one and two weeks post-transduction (Figure 6E). Two-week lentiviral MGT transduction was associated with similar apoptosis- and necroptosis-associated responses in primary BMDMs, with total MLKL levels also significantly elevated (Figure S1). Together, these findings support the activation of a necroptosis-associated pathway in MGT-transduced BMDMs, in addition to the apoptosis-associated responses described above.

## 4. Discussion

In this study, we first established and validated a polycistronic lentiviral MGT system in cardiac fibroblasts, confirming its expected induction of cardiomyocyte-associated molecular responses. When applied to macrophages, the MGT regimen induced an early cardiac-associated transcriptional response, including increased expression of Tnnt2 and Myh6, together with increased cTnT protein expression. However, these changes were incomplete and were not accompanied by a convincing cardiomyocyte phenotype, indicating that MGT exposure elicited a partial cardiac-associated response rather than efficient reprogramming. The broader plasticity of macrophages provides some biological rationale for exploring this possibility. During fibrosis, macrophages can undergo MMT, while previous studies have shown that myofibroblasts may be reprogrammed into cardiomyocytes [31,32,33,34]. However, these observations do not establish that macrophages themselves can be directly converted into cardiomyocytes. Although macrophages are separated from cardiomyocytes by a considerable lineage distance, the induction of cardiac marker genes such as *Tnnt2* and *Myh6* by MGT suggests that at least some cardiomyocyte-associated transcriptional programs are accessible to MGT-mediated activation in macrophages. Whether additional chromatin-remodeling or epigenetic strategies could enhance this partial response remains to be determined. It is important to note that BMDMs may not fully recapitulate the response of resident cardiac macrophages in vivo. Resident cardiac macrophages are developmentally and transcriptionally distinct from monocyte-derived macrophages and are adapted to the cardiac niche. Their pre-existing adaptation to the cardiac environment may therefore influence their response to MGT exposure.

Our data further demonstrated that MGT treatment was associated with a shift toward an M2-like macrophage state, as indicated by the sustained upregulation of Cd206 expression. Macrophages are highly plastic cells whose phenotypic states are shaped by the microenvironment and dynamically change during myocardial infarction. During the post-MI reparative phase, the transition toward an M2-like state is generally associated with resolution of inflammation and tissue repair, with M2-like macrophages, including CCR2^−^ resident cardiac macrophages, contributing to tissue remodeling [35]. Thus, the MGT-induced M2-like shift could initially appear favorable in the context of cardiac repair. However, macrophage states are highly heterogeneous, and increased Cd206 expression alone does not establish a uniformly reparative phenotype. Importantly, the post-MI resolution phase is also characterized by increased TGF-β signaling, which promotes fibroblast-to-myofibroblast conversion and extracellular matrix deposition [36,37,38], and has been reported to promote the transition of M2a macrophages toward myofibroblasts through MMT [39]. Although we did not directly assess MMT in this study, the MGT-induced shift toward an M2-like state raises the possibility that MGT-exposed macrophages could become more susceptible to TGF-β-driven MMT under the post-MI microenvironment. Thus, the consequences of MGT exposure may depend on the balance between its effects on macrophage polarization and cell survival, as well as the timing and cellular context of exposure. Whether these effects ultimately promote inflammatory resolution and tissue repair or contribute to adverse remodeling and fibrosis warrants further investigation in vivo.

MGT treatment induced substantial apoptosis-associated and necroptosis-associated responses in primary BMDMs, consistent with previous reports that ectopic expression of reprogramming factors can impose cellular stress [40,41,42,43]. Importantly, these responses were observed following lentiviral MGT transduction, rather than direct plasmid transfection, suggesting that they cannot be readily attributed solely to exogenous plasmid DNA exposure. These findings suggest that vector selection alone may not eliminate the safety concerns associated with ectopic MGT expression, and that the biological consequences of ectopic MGT expression in primary macrophages warrant careful consideration in the development of in situ reprogramming strategies. This also raises the question of whether the adverse effects of MGT exposure on non-target cardiac cells could be mitigated. Neuregulin-1 has been implicated in cardiovascular protection through multiple mechanisms, including activation of pro-survival signaling pathways such as PI3K-AKT, and may therefore represent a potential strategy for mitigating reprogramming-associated cellular stress [44]. Whether NRG1 or related protective approaches can reduce MGT-associated macrophage dysfunction or cell-death-associated responses without compromising cardiac reprogramming efficiency remains to be determined.

Several limitations should be acknowledged: 1) this study was performed in cell culture models, and the observed responses may not fully recapitulate the behavior of macrophages in the cardiac microenvironment; 2) we did not evaluate MGT exposure in a disease-relevant in vivo setting; and 3) we did not investigate the influence of macrophage heterogeneity, particularly whether resident cardiac macrophages differ from infiltrating monocyte-derived macrophages in their responses to MGT. These questions warrant further investigation.

## Data Availability

The datasets used and/or analyzed during the current study are available from the corresponding author upon request.

## Supplementary Materials

The following supporting information can be downloaded at https://www.mdpi.com/article/10.3390/biomedicines14092089/s1, Figure S1: Two-week Lentiviral MGT transduction induces apoptosis- and necroptosis-associated responses in primary BMDMs; Table S1: List of primers used for this study.

## Abbreviations

The following abbreviations are used in this manuscript:

LVs	lentiviruses
CFs	cardiac fibroblasts
MGT	Mef2c, Gata4, and Tbx5
iCMs	induced cardiomyocytes
BMDMs	bone marrow-derived macrophages
CVDs	Cardiovascular diseases
MI	myocardial infarction
MMT	macrophage-myofibroblast transition
