# Supplementary material for: Macrophage Mistargeting During Direct Cardiac Reprogramming: Cardiac-Associated Responses, Polarization, and Cell Death
Source: Biomedicines. 2026 Sep 16;14(9):2089. doi: 10.3390/biomedicines14092089 (PMC13604069; doi:10.3390/biomedicines14092089)
Supplement: Supplementary file 1 [file biomedicines-14-02089-s001.zip › Figure S1.pdf]

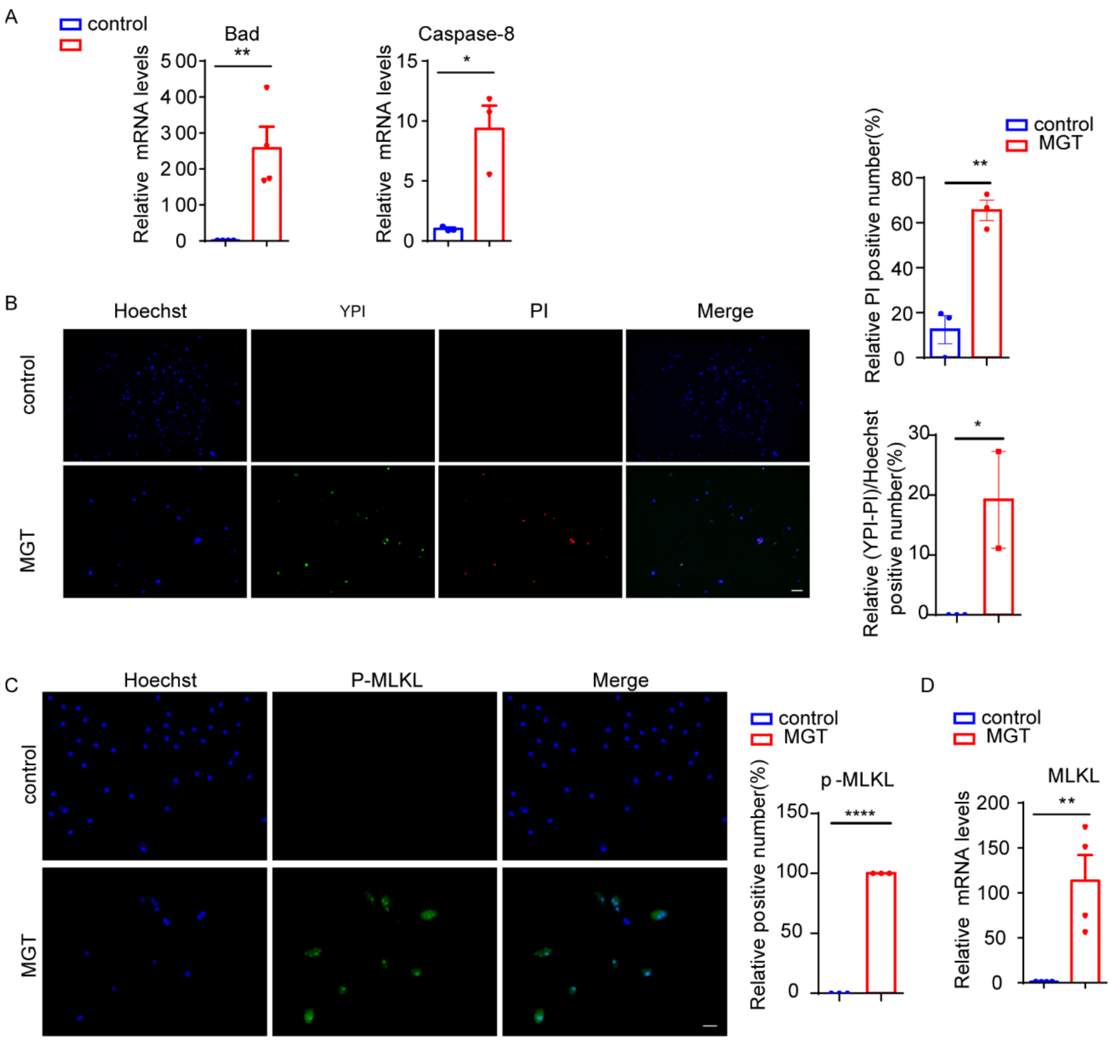

Figure S1. Two-week Lentiviral MGT transduction induces apoptosis- and necroptosis-associated responses in primary BMDMs (A) Bar graphs (Mean + SEM) overlaid with dot plots (individual data points) showing the corresponding mRNA levels of *Bad, Caspase-8* and *MLKL* evaluated by qPCR (n = 3-4). (B) YP1-PI staining. Scale bar = 20 μm. (C) Primary BMDMs were transduced by the direct lentiviral plasmid transfection overexpressing MGT for a week. Immunofluorescence for P-MLKL (n =2-3). Scale bar = 20 μm. (D) MRNA levels of *MLKL* evaluated by qPCR (n = 4). $*p < 0.05$, $**p < 0.01$, $***p < 0.001$, $****p < 0.0001$. Unpaired Student's t-test.
