# Supplementary material for: Macrophage Mistargeting During Direct Cardiac Reprogramming: Cardiac-Associated Responses, Polarization, and Cell Death
Source: Biomedicines. 2026 Sep 16;14(9):2089. doi: 10.3390/biomedicines14092089 (PMC13604069; doi:10.3390/biomedicines14092089)
Supplement: Supplementary file 1 [file biomedicines-14-02089-s001.zip › Table S1.pdf]

Supplementary Tables:

Supplementary Table S1: List of primers used for this study.

**Gene Name**	**Forward Primer(5'-3')**	**Reverse Primer(5'-3')**
Mef2c	ATCCCGATGCAGACGATTCAG	AACAGCACACAATCTTTGCCT
Gata4	CCCTACCCAGCCTACATGG	ACATATCGAGATTGGGGTGTCT
Tbx5	ATGGCCGATACAGATGAGGG	TTCGTGGAACTTCAGCCACAG
Tnnt2	CAGAGGAGGCCAACGTAGAAG	CTCCATCGGGGATCTTGGGT
Myh6	GCCCAGTACCTCCGAAAGTC	GCCTTAACATACTCCTCCTTGTC
Ryr2	ACGGCGACCATCCACAAAG	AAAGTCTGTTGCCAAATCCTTCT
CD68	TGTCTGATCTTGCTAGGACCG	GAGAGTAACGGCCTTTTTGTGA
iNOS	CAGATCGAGCCCTGGAAGAC	CTGGTCCATGCAGACAACCT
CD206	CTCTGTTCAGCTATTGGACGC	TGGCACTCCCAAACATAATTTGA
GAPDH	AGGTCGGTGTGAACGGATTTG	TGTAGACCATGTAGTTGAGGTCA
Bad	AAGTCCGATCCCGGAATCC	GCTCACTCGGCTCAAACTCT
Caspase-8	TGCTTGGACTACATCCCACAC	TGCAGTCTAGGAAGTTGACCA
Bcl2	GTCGCTACCGTCGTGACTTC	CAGACATGCACCTACCCAGC
MLKL	AATTGTACTCTGGGAAATTGCCA	TCTCCAAGATTCCGTCCACAG
